# Comparison of the Automatic and Manual Broiler Pre-Slaughter Chain Based on Trailer Microclimate during Transportation and Its Effect on *m*. *pectoralis major*

**DOI:** 10.3390/ani11102946

**Published:** 2021-10-12

**Authors:** Filip Beňo, Tereza Škorpilová, Václav Pohůnek, Josef Bauer, Rudolf Ševčík

**Affiliations:** Department of Food Preservation, Faculty of Food and Biochemical Technology, University of Chemistry and Technology, Prague, Technická 5, 166 28 Prague, Czech Republic; tereza.skorpilova@vscht.cz (T.Š.); vaclav.pohunek@vscht.cz (V.P.); josef.bauer@vscht.cz (J.B.); rudolf.sevcik@vscht.cz (R.Š.)

**Keywords:** broiler, pre-slaughter handling, welfare, meat quality, pH, EC, lightness

## Abstract

**Simple Summary:**

Improper pre-slaughter catching, loading/unloading, handling, and transport may cause negative effects on the welfare and meat quality of poultry. During the catch process, noisy, rough, or aggressive techniques can cause birds to panic, which can lead to injuries and lower meat quality. Fractures, joint dislocations, and bruises can be common and cause bird suffering, mortality, carcass degradation, and economic loss. Proper pre-slaughter processes can ensure the safety of poultry and workers. One way to prevent these problems is to use automatic catching machines (harvesters/chicken cat), handling machines (shuttles), and air-conditioned trucks (trailers) to collect and handle poultry. Microclimate in trailers is another important factor influencing welfare. Internal overheating may cause high mortality of the animals during transport and reduced meat quality. The use of modern air-conditioned trailers results in improved welfare conditions, reduced mortality and the incidence of injuries and fractures, and increased meat quality.

**Abstract:**

This study aims to compare two broiler pre-slaughter chain methods: (i) the automatic pre-slaughter chain (APC) and (ii) manual pre-slaughter chain (MPC). The comparison is based on the evaluation of the trailer microclimate, number of injuries, and breast muscle (*m. pectoralis major*) quality. Transportation lasts 3.5 h, unloading 1 h. The selection of two hundred 39-day-old broilers (Ross 308 and Cobb 500 breeds) is random for each type of method. After slaughter, the pH value, electrical conductivity (EC), and color (lightness) of breast muscle tissues are determined at different post-mortem intervals. The MPC negatively affects the microclimate (*p* < 0.001), meat qualitative characteristics (*p* < 0.001), and places a greater strain on the body of chickens compared with APC. The average pH_15min_ value of MPC broiler breast muscle tissue, generally used as the main meat quality parameter, is 5.97 ± 0.12, in contrast to 6.36 ± 0.16 for APC. Higher pH_15min_ value of APC indicates better welfare and pre-slaughter handling. Values of EC and L* of breast tissues also confirms a difference between the methods of broiler handling (*p* < 0.001). No difference is found between the breed lines (*p* > 0.05).

## 1. Introduction

Considering the potential impact on the welfare and thus meat quality of poultry, pre-slaughter handling is considered a key point of poultry production [1]. Poultry is handled, transported, and slaughtered in higher numbers than any other livestock. In comparison to other farm animals, poultry has a shorter generation interval and a higher feed conversion ratio. Many operations in the broiler production chain are being criticized in terms of animal welfare as mass production can result in violations of animal welfare guidelines [2,3,4,5]. Pre-slaughter operations can result in bird fear, stress, pain, injury, and higher mortality [4,6,7]. In reality, poultry are subjected to a variety of stressors, including heat/cold stress and extended travel distances. Thermal stress, whether it is extreme heat or extreme cold, has a negative impact on poultry welfare during transportation [3]. The climatic conditions in crates and trailers are frequently overlooked while transporting poultry [8]. Poor welfare quality related to morbid conditions during transportation, caused by the thermal environment and pressure of transportation (air movement, low ambient temperature, and winter precipitation) and the decline in meat quality of slaughtered birds, cause big economic losses [9,10]. Stressful events (as well as interactions between animals and humans) trigger reactions in animals that translate into physical, physiological, and behavioral stress [10,11]. Although the genetic selection of broilers has greatly improved the growth rate and production efficiency, these advances may also be related to the reduced resistance to heat or cold stress and muscle pathology. The result of all these pressures may reduce the welfare and quality of poultry meat and may result in PSE (pale, soft, and exudative characteristics) meat conditions. Lower pH, higher EC, higher lightness, softer texture, lower water holding capacity, and higher yield loss are characteristic for PSE-like meat [11,12].

Broiler normal pH_15min_ is about 6.2 to 6.5 and the final (pH_24h_) is about 5.8 post-mortem [13,14,15]. Most authors agree that the pH_15min_ of PSE-like meat is pH < 5.8 [16,17,18]. The measurement of muscle tissue electrical conductivity (EC) can also be used to assess the quality of meat because EC values correlate with the muscle condition deviation similarly to PSE-like meat. In addition, there is a negative correlation between pH and EC. Due to high temperature and decreasing pH during the intensive glycolysis that takes place post-mortem, cell membranes and walls in the PSE-like muscles are disturbed; the intracellular fluid containing ions leaks out and the value of EC of muscle grows. After stunning, the pH value decreases while EC grows [19,20]. Flesh color is an important criterion that affects consumer choice and an important characteristic of meat quality.

The discoloration of broiler meat is closely related to the post-mortem decrease of muscle pH value. Lower pH_15min_ (<5.8) and near to the isoelectric point leads to a discoloration of muscle tissue [13]. The criteria values of lightness (L*), determining meat quality and PSE-like meat presence, are based on studies where L*_24h_ > 53 were classified as PSE and normal 46 < L*_24h_ < 53 [21,22], or lightness with 50 ≤ L*_1h_ ≤ 56 are classified as normal and L*_1h_ > 56 as PSE-like meat [16].

The most likely causes of these problems are the poor design of transport crates and vehicles with not fully controllable temperature, relative humidity, and air-flow inside the cargo bay conditions [6,23]. Current manual pre-slaughter handling involves bird-catching, placing in crates, and then loading the crates into vehicles or trailers to transport the birds to the slaughterhouse [7,24]. To avoid causing extra discomfort and bad welfare conditions to the poultry, pre-slaughter methods must be developed and managed. One option is to use automatic handling of poultry during pre-slaughter processes [4,5].

Automatic pre-slaughter methods are already used in countries such as the Netherlands and Belgium [4,5]. In the Czech Republic, this method was first used in 2016, and is called the peer system. The technology of the peer system consists of automatic bird catching machines (harvesters), handling machines (shuttles), and trucks (trails) equipped with ventilation. Ventilation guarantees sufficient air circulation and an efficient air-conditioning system. The principal structure of the machine consists of a collecting head system operated by hydraulics and a conveyor belt used to place the broilers on one of the peer system shuttles. The peer system trailer consists of ten moving floors that gently bring the broilers in/out. One possible explanation for the reduction of stress is that the birds remain upright and do not stand upside down during mechanical capture.

In this study, two broiler pre-slaughter chains are compared: (i) the automatic pre-slaughter chain (APC) and (ii) manual pre-slaughter chain (MPC). To compare the two methods, the microclimate during transport, the incidence of broiler carcass injuries, and the quality of the *pectoralis major* muscle are assessed.

## 2. Materials and Methods

### 2.1. Broiler Loading, Transport, and Unloading

**Figure 1:**
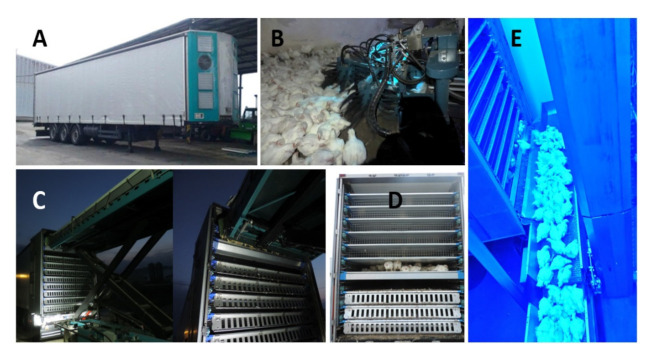
Automatic poultry pre-slaughter machines. (**A**) Trailer equipped with ventilation; (**B**) harvesters; (**C**) shuttles; (**D**) trailer floors; (**E**) automatic conveyor belt.

The experiment took place in March 2020 and the average temperature of the month was 5.7 °C, with an average relative humidity of 54%. The average temperature of the experiment day was 3.0 °C, with an average relative humidity of 57%. The lowest temperature of the day was −5.0 °C and the highest was 10.4 °C, with no rainfall. Broiler loading occurred on the same day in one farm from 2 halls (South Bohemia, Czech Republic). There was a broiler genotype in each hall. Loading began at 2:45 a.m. and lasted 1.5 h until both types of trailers were filled. In each hall, the loading was done simultaneously, using both the automatic method and the manual method. In a conventional trailer, broiler genotypes were divided into right and left sides. The catching of the chickens has been set so that the density of birds in both type trailers is 32 kg/m^2^, which corresponds to about 14–16 birds/m^2^. For the peer system, genotypes were divided into odd and even floors. Broiler loading was followed by a 0.5 h rest. At 4:45 a.m., both trucks (APC and MPC) left for the slaughterhouse. The transport took a total of 3.5 h. This was followed by a further 0.5 h rest. After unloading the broilers, 100 of each genotype were randomly selected after stunning. Figure 1 shows the automatic pre-slaughter machines—“the peer system”.

### 2.2. Trailer Microclimate Measurement

**Figure 2:**
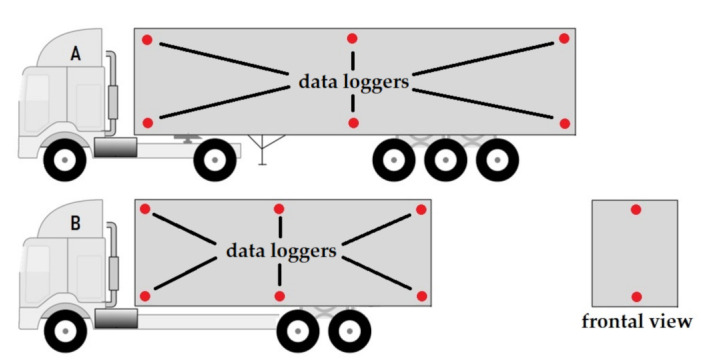
Trailer schemes (**A**–APC, **B**–MPC) with data logger locations. The data loggers were equally distributed in the top and bottom of each trail section (front, center, and rear).

To record the temperature and relative humidity in trailers, data loggers (Thermochron iButton DS1922L, MSL, San Jose, PA, USA) were used. Temperature was recorded in 10 min intervals. The QiTerm software was used for evaluation. There were 6 sensors placed in each truck trailer before the transport began; for the exact positions of the data loggers, see Figure 2A,B.

### 2.3. Samples

At 39-days-old, two hundred broilers from Cobb 500 (*n* = 100) and Ross 308 (*n* = 100) breeds, raised in an intensive system in the same microclimate and feeding conditions, were collected randomly from both pre-slaughter methods, the automatic pre-slaughter chain (APC) and manual pre-slaughter chain (MPC). In total, four groups were made (4 × *n* = 50): (i) Cobb from MPC, (ii) Ross from MPC, (iii) Cobb from APC, and (iv) Ross from APC. Broilers were stunned in an electric water bath. The stunning intensity of 120 mA with a frequency of 50 Hz for 4–6 s was used. The average slaughter weight of the broilers was 2.19 ± 0.43 kg. After stunning, killing by decapitation, bleeding, plucking, and gutting, carcasses were immediately stored in the refrigerator at 4 °C for 24 h.

### 2.4. pH Measurement

Breast muscle tissue pH value was measured by the direct probe method using a needle pH meter (Matthäus, Pöttmes, Germany). Values of pH (pH_15min_, pH_4h_, pH_24h_) were taken from the left and right breast muscle tissue post-mortem. The electrode was inserted approximately 5 cm deep into the breast tissue (*pectoralis major* muscle). Furthermore, the temperature was measured using a thermometer (Testo 110, Testo AG, Lenzkirch, Germany) with a temperature probe (Testo GmbH & Co., Testo AG, Lenzkirch, Germany) (range from −50 to 150 °C) before each pH measurement. The measured temperature was adjusted with a pH meter for correlation.

### 2.5. Electrical Conductivity Measurement

The tissue electrical conductivity was measured using the LF STAR (Matthäus, Pöttmes, Germany) with two parallel steel electrodes. It was measured at 1 h (EC_1h_), 4 h (EC_4h_), and 24 h (EC_24h_) post-mortem at both breast muscles about 3 cm deep in the tissue. The device was calibrated to 10 mS·cm^−1^.

### 2.6. Color Measurement

Broiler breast meat color was measured by reflectance of tissue with a spectrophotometer (Minolta CM–2600D, Tokyo, Japan) using CIE L* (lightness), CIE a* (redness), and CIE b* (yellowness). The device was calibrated to white (L* = 100) and black (L* = 0) colors. The parameters of the instrument are: mask–MAV (8 mm), gloss–SCE, UV–100% Full, observer–10°, illuminant D 65, color space–CIE L*a*b*. To prove PSE presence, only the CIE L* values were used.

### 2.7. Statistics

The data collected were used for statistical evaluation. The variables were muscle pH value, electrical conductivity, and color/lightness. The statistical significance between transport types and breed was statistically determined by the method PCA (Principal Component Analysis) created in the software STATISTICA 12.0 CZ (StatSoft, Prague, Czech Republic). Mixed-design ANOVA was applied to collected data. The pre-slaughter chain and breed line were considered to be a fixed effect, and pH value, EC, and L* were included in the model as a random effect (MIXED procedure). The differential analysis was performed using a t-test and the *p*-Value with 95% confidence interval.

## 3. Results

The transportation microclimate was recorded and post-mortem changes were controlled by measuring the qualitative characteristics of the breast muscle (*pectoralis major*). The variations of the pH value, electrical conductivity, and lightness of breast muscle tissue by transport and microclimate conditions of transport are given in Table 1. Pre-slaughter chain methods differed (*p* < 0.001) in every characteristic.

**Figure 3:**
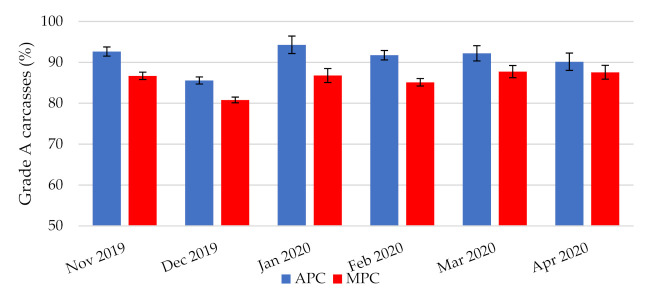
Grade A of carcass representation (*n* = 20,754 for APC; *n* = 19,345 for MPC).

**Figure 4:**
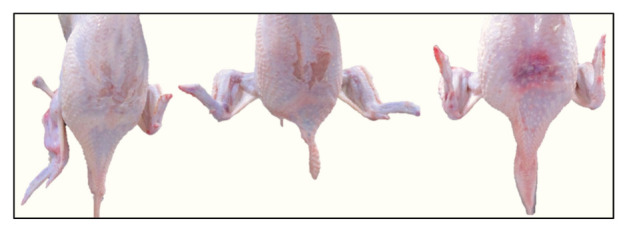
Grade B of broilers with bruises, skin injuries, and fractures.

In Figure 3, the distribution of broiler carcasses after meat processing is shown by two grades, “A” and “B”. Grade “A” demands good body structure and meaty, with no bruises, no fractures, and a thin layer of fat under the skin. The carcasses with bruises, abrasions, fractures, etc. are classified as grade “B” (Figure 4).

**Figure 5:**
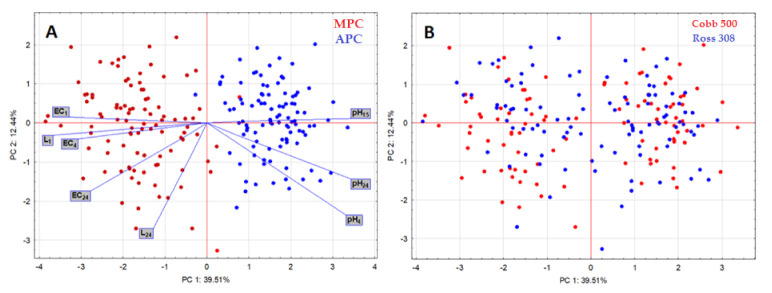
2–D plots of principal component analysis (PCA) of the qualitative characteristics of transported broilers. Distribution of (**A**) pre-slaughter MPC and APC chain and (**B**) breed lines Cobb 500 and Ross 308.

For a comparison of the breed line (Cobb 500 and Ross 308) results, see Table 1. The difference was noted only for the measurement of electrical conductivity EC_24h_. The difference between pre-slaughter methods was also confirmed by the PCA analysis (Figure 5A). Breed × pre-slaughter method was not found to be significant (Figure 5B).

## 4. Discussion

Transport, poultry handling, and poultry slaughter technology alone have an impact on the quality of poultry meat produced. Any stress effects resulting in a reduction in the quality of the meat must be minimized. Poultry is most exposed to the stress of being caught on farms, transported, and then taken to the slaughterhouse and hooked before stunning [7,25]. The warm summer months are particularly problematic during transport, when high temperatures during transport can lead to overheating of poultry and subsequent death during transport or deterioration of meat quality. The aim of each meat-processing food business operator should be to improve the welfare of the animals and minimize the losses associated with the culling of poultry and the reduction in the quality of the meat produced. Increasing the level of welfare is possible by removing broilers from farms and transported to slaughterhouses using automatic pre-slaughter methods. Transportation in crates is used only on small farms where it is not possible to use the peer loading system because of inadequate farm space.

From the data from this study, it has been shown that the APC positively influences the welfare and quality of slaughtered broiler meat. Data presented by Nijdam et al. [26] show different results. In their study it was observed that automatic catching causes slightly higher mortality rates and for meat quality and stress, no differences were found between automatic and manual catching of broilers. In our study, the most important significant difference was observed in microclimate conditions (*p* < 0.001), as also shown by the high temperature and relative humidity standard deviations, and the maximum and minimum values (Table 1). The experiment in our study was carried out in March 2020. The average temperature of the day (time of day during the transportation) was 12.8 ± 2.3 °C and the average relative humidity was 57.1 ± 3.51 %. The temperature in the case of MPC fluctuated considerably. MPC might have had a negative impact on the meat quality and welfare of broilers. This fact is caused by fluctuating temperature, relative humidity, and lack of space and air during transport, causing stress to the poultry. Under optimal conditions, broilers should be exposed to a temperature in the so-called thermoneutral zone during transport. The results of our study do confirm previous research by Webster et al. [27], in which the optimal thermal zone is 8–18 °C. In contrast to the earlier findings of Meltzer [28], these results suggest an optimal thermal zone from 24 to 28.5 °C. Temperature fluctuations and relative humidity may be a particular problem causing higher mortality and frequent injuries of transported broilers [7,9,29,30,31].

From the data collected during the half-year period (November 2019 to April 2020), it was found that there was an increase in the prevalence of grade “A” broiler carcasses due to the new automated pre-slaughter chain (Figure 3). Automatic catching, loading, transporting, and unloading the broilers caused fewer injuries compared to the manual method. Similar results were obtained in the experiment by Knierim and Gocke [32]. The number of grade “A” carcasses has been determined using image analysis, which is placed in front of the cutting room. The representation of Grade “A” between the APC and MPC method is different across several months (*p* = 0.002). Average increase of grade “A” carcasses was about 5.3%.

The average pH values after 15 min, 4 h, and 24 h of the breast muscles were lower in the MPC broilers compared to APC broilers (*p* < 0.001), versus the values of electrical conductivity and lightness of MPC breast muscle were higher compared with APC after 1, 4, and 24 h post-mortem. Lower average pH_15min_ value of MPC broiler breast muscles approaching the limit of 5.8 (according to literature reports) indicates a lower quality of the meat [16,17,18]. Contrary to what has been reported by Warris et al. [33] and Kannan et al. [15], our results indicate that APC and MPC influence pH value of broiler breast muscle. According to Ingr et al. [19], Saelin et al. [20], Byrne et al. [34], and Ovchynnikova et al. [35], negative correlations between pH and EC values of MPC (r = −0.81) and APC (r = −0.92) broiler breast muscle tissues were found. As mentioned by Saelin et al. [20], lower pH values lead to higher electrical conductivity values.

Referring to Ingr et al. [19], Šimek et al. [36], Byrne et al. [34], Thielke et al. [37], and our collected pH values, EC_1h_ < 5 mS·cm^−1^ was classified as high-quality meat, 5 < EC_1h_ < 9 mS·cm^−1^ as reduced quality meat, and EC_1h_ > 9 mS·cm^−1^ correspond to PSE-like meat. Although the average EC_1h_ value is not below the limit of 5 mS·cm^−2^, taking the standard deviation into account, the quality of the MPC meat is inclined to be of reduced quality, but not of PSE-like meat.

In the current study, the L* (1 and 24 h after slaughter) average value of the breast muscles of the APC broilers was higher compared with MPC broilers. Lightness is highly correlated with muscle tissue pH value and lower pH results in higher L* values [38,39]. The average L*_1h_ value of MPC indicates PSE-like meat according to Petracci et al. [16] and L*_24h_ values [21,22] also confirmed this fact. However, pH values did not confirm PSE meat conditions. Therefore, lightness is not an appropriate indicator for the determination of broiler breast meat quality muscle based on Molette et al. [40] and our data. The problem is also the character of broiler breast muscle, due to low myoglobin level and the presence of oxygen, causing pale meat color.

According to Table 1, there are no significance differences between breed lines (*p* > 0.05), except for values of electrical conductivity 24 h post-mortem (*p* < 0.05). As determined by Glamoclija et al. [41], pH values 15 min after the slaughter of 42-day-old different broiler breed lines were 6.44 for Cobb and 6.29 for Ross. In the current study, pH_15min_ values for both breed lines were lower and similar for both breed lines, 6.14 for Cobb 500 and 6.18 for Ross 308. Values of pH taken 24 h (6.05 for Cobb 500 and 6.07 for Ross 308) after slaughter were similar just for Cobb breed lines, compared to Glamoclija et al. [41] (6.04 for Cobb 500 and 6.07 for Ross 308). Breed lines had no impact on the qualitative characteristics of *pectoralis major* of broilers.

The data obtained from the qualitative characterization of broiler breast muscles were subjected to principal component analysis (PCA), as shown in Figure 5. The first two components of the ordination explained 51.97% of the variation, with 39.51 and 12.44% for PC1 and PC2. The peer system clearly separated from conventional transport (Figure 5A). The peer system transport is closely correlated with pH values, versus conventional transport with EC and L* values. There was no important difference between the breed lines (Figure 5B).

## 5. Conclusions

Pre-slaughter chain influences broiler meat quality because broilers are subjected to the greatest stresses associated with environmental changes and may cause broiler meat condition defect (PSE), skin injuries, and limb fractures, thereby affecting the loss of raw materials. The results of our study indicate that MPC affected the *m. pectoralis major* and broiler welfare negatively. The trailer microclimate, temperature, and relative humidity is controlled during APC, leading to better well-being of transported broilers, with no overheating, overcooling, or gasping for breath. The introduction of the new peer system has also led to a reduction in the risk of injury and fractures (5–6%). MPC broiler resulted in lower pH values, higher electrical conductivity, and higher lightness of breast tissues. Breed lines had no impact on the qualitative characteristics of *m. pectoralis major* of the broilers.

## Figures and Tables

**Table 1:** Qualitative characteristics of *m. pectoralis major* of broilers and the trailer microclimate conditions (mean ± SD).

Qualitative Characteristics	*n*	Pre-Slaughter Chain			Breed Line			ANOVA
		MPC	APC	*p*-Value	Cobb 500	Ross 308	*p*-Value	*p*-Value Pre-Slaughter Chain × Breed
pH_15min_	100	5.97 ± 0.12	6.36 ± 0.17	<0.001	6.14 ± 0.24	6.18 ± 0.23	0.654	0.8358
pH_4h_		5.83 ± 0.19	6.01 ± 0.14	<0.001	5.91 ± 0.19	5.93 ± 0.18	0.874	0.0379
pH_24h_		5.94 ± 0.16	6.16 ± 0.15	<0.001	6.05 ± 0.19	6.07 ± 0.17	0.794	0.2902
EC_1h_ (mS·cm^−1^)	100	4.64 ± 0.81	3.49 ± 0.67	<0.001	3.99 ± 0.86	4.14 ± 1.01	0.283	0.2880
EC_4h_ (mS·cm^−1^)		4.95 ± 0.87	3.86 ± 0.52	<0.001	4.46 ± 0.95	4.34 ± 0.83	0.397	0.1195
EC_24h_ (mS·cm^−1^)		4.29 ± 0.93	3.66 ± 0.61	<0.001	4.11 ± 0.89	3.84 ± 0.78	<0.05	<0.0001
L*_1h_	100	56.65 ± 3.25	52.20 ± 3.16	<0.001	54.19 ± 3.70	54.66 ± 4.07	0.595	0.6050
L*_24h_		54.83 ± 2.64	52.86 ± 2.37	<0.001	54.00 ± 2.63	53.68 ± 2.75	0.690	0.0283
Trailers microclimate	*n*	Min	Max	Ø MPC	Min	Max	Ø APC	*p*-Value
T (°C)	6	2.6	25.8	12.9 ± 5.6	17.1	23.7	18.7 ± 1.8	<0.001
RH (%)		30.7	73.5	54.3 ± 20.1	38.4	64.2	51.1 ± 11.3	<0.001

MPC = manual pre-slaughter chain; APC = automatic pre-slaughter chain; pH_15min_ = pH 15 min after slaughter; pH_4h_ = pH 4 h after slaughter; pH_24h_ = pH 24 h after slaughter; EC_1h_ = electrical conductivity 1 h after slaughter; EC_4h_ = electrical conductivity 4 h after slaughter; EC_24h_ = electrical conductivity 24 h after slaughter; L*_1h_ = lightness 1 h after slaughter; L*_24h_ = lightness 24 h after slaughter; Ø = average value; T = temperature; RH = relative humidity; *n* = number of samples/data loggers.

## Data Availability

Data available on request due to privacy restrictions. The data presented in this study are available on request from the corresponding author.
